# Application of Circulating Tumor Cells and Circulating Free DNA from Peripheral Blood in the Prognosis of Advanced Gastric Cancer

**DOI:** 10.1155/2022/9635218

**Published:** 2022-01-11

**Authors:** Pengjie Yu, Shengmao Zhu, Yushuang Luo, Ganggang Li, Yongqiang Pu, Baojia Cai, Chengwu Zhang

**Affiliations:** ^1^Department of Gastrointestinal Oncology, Affiliated Cancer Hospital of Qinghai University, Affiliated Hospital of Qinghai University, Xining, China; ^2^Internal Medicine-Oncology, Affiliated Cancer Hospital of Qinghai University, Affiliated Hospital of Qinghai University, Xining, China; ^3^The Fifth People's Hospital of Qinghai Province, Xining, China

## Abstract

**Objective:**

To explore the application value of circulating tumor cells (CTCs) and circulating free DNA (cfDNA) from peripheral blood in the prognosis of advanced gastric cancer (AGC). Here, we measured CTCs and cfDNA quantity for predicting the outcome of patients. *Patients and Methods*. Forty-five patients with advanced gastric cancer who underwent neoadjuvant chemotherapy and surgical treatment were enrolled in this study. All patients received neoadjuvant chemotherapy with paclitaxel + S-1 + oxaliplatin (PSOX) regimen, and CTCs and cfDNA of the peripheral blood were detected before and after neoadjuvant therapy. Relationships between the number/type of CTC or cfDNA and the efficacy of neoadjuvant chemotherapy were analyzed.

**Results:**

Among 45 patients, 43 (95.6%) were positive, and the positive rate of mesenchymal CTC was increased with the increase in the T stage. The proportion of mesenchymal CTC was positively correlated with the N stage (*P* < 0.05), and the larger N stage will have the higher proportion of mesenchymal CTC. Patients with a small number of mesenchymal CTC before neoadjuvant chemotherapy were more likely to achieve partial response (PR) with neoadjuvant therapy. Patients with positive CA-199 were more likely to achieve PR with neoadjuvant therapy (*P* < 0.05). Patients in the PR group were more likely to have decreased/unchanged cfDNA concentration after neoadjuvant therapy (*P*=0.119). After neoadjuvant therapy (before surgery), the cfDNA concentration was higher and the efficacy of neoadjuvant therapy (SD or PD) was lower (*P*=0.045).

**Conclusions:**

Peripheral blood CTC, especially interstitial CTC and cfDNA, has a certain value in predicting the efficacy and prognosis of neoadjuvant chemotherapy in advanced gastric cancer.

## 1. Introduction 

Gastric cancer (GC) is one of the most common malignant tumors of the digestive tract according to World Health Organization (WHO) data [1]. Worldwide, the incidence of gastric cancer is 13.86 per 100,000 people [2]. Gastric cancer in China has a high mortality rate and is up to 20/100,000 [3]. Most case belong to advanced gastric cancer (AGC) based on standard tumor-node-metastasis (TNM) staging [4] when they were diagnosed in China. Surgery of no doubt is the best treatment tool for those who were classified as highly differentiated GC. However, the number of AGC patients for surgery was limited because of their staging. Recently, many studies suggested that patients with cancer can perform preoperative or perioperative neoadjuvant chemotherapy for shrinking tumor size or killing micrometastases [5–7]. This definitely increased successful chances for surgery. Therefore, the key for the treatment of the patients with GC is to identify sensitivity and specificity markers at their early stage. With the application of liquid biopsy technology, circulating tumor cell (CTC) and circulating free DNA (cfDNA) have been used in effect evaluation of clinical tumor treatment and recurrence risk detection [8–10]. CTCs are cells that release into the blood stream from the primary tumor site. CTCs can become seeds of metastasis in distant organs and drive cancer to relapse [7]. CTCs are divided into epithelial, mesenchymal, and mixed CTCs based on their cell surface markers, in which epithelial CTCs are characterized with EpCAMplus CK8/18/19 and mesenchymal CTCs mark vimentin and twist, respectively [11, 12]. The measurement of CTCs from peripheral blood in cancer patients is used in the detection of breast cancer, bladder cancer, nonsmall cell lung cancer, and other solid tumors [13–16]. Circulating free DNA (cfDNA) is about 50–200 base pair (bp) length DNA fragment and can freely circulate in the bloodstream [17]. CfDNA may be from the cellular nucleus or mitochondria and has a specific genetic mutation or epigenetic abnormal information. These aberrant genetic materials can be used for diagnosis and predicting the prognosis of the disease [18]. Many studies revealed that cfDNA levels of patients with advanced-stage cancer were elevated [19–21]. However, CTCs and cfDNA levels of patients with advanced gastric cancer are limited. This study aimed to analyze the levels and types of peripheral blood CTC and cfDNA in patients with advanced gastric cancer during perioperative treatment. We also evaluated the application value of peripheral blood CTC and cfDNA in the outcomes of advanced gastric cancer.

## 2. Materials and Methods

### 2.1. Subjects

The patients with advanced gastric cancer (AGC) who had undergone neoadjuvant chemotherapy and surgery in the Department of Gastrointestinal Surgery of the Affiliated Hospital of Qinghai University between September 2019 and October 2020 were enrolled in this study. Enrollment criteria were as follows: (1) a total of 45 cases were diagnosed with gastric cancer by endoscopy and tumor tissues by biopsy; (2) TNM staging of all patients was T3-4N × M0 (according to the TNM staging standard of gastric cancer AJCC/UICC 8th edition, staging is mainly based on abdominal CT, combined with gastroscopy, B ultrasound, etc., if necessary, ultrasound endoscopy, MRI, etc.); (3) physical status score of eastern cooperative oncology group (ECOG) ≤2 points and could tolerate chemotherapy; (4) newly diagnosed patients with no previous radical or palliative surgery, radiotherapy, and chemotherapy history; (5) the functions of liver and kidney were in the normal range; and (6) age was between 18 and 80 years old. Exclusion criteria were as follows: (1) patients with pyloric obstruction, upper gastrointestinal hemorrhage, gastrointestinal perforation, severe infection, and other complications; (2) existed history of radical or palliative surgery, radiotherapy, and chemotherapy or biological therapy; (3) history of allergy to chemotherapy drugs; (4) pregnant or breastfeeding; (5) patients with distant metastases; and (6) other malignant tumors. In this study, 45 patients with advanced gastric cancer were enrolled, including 40 men, 5 women, and age of 29–69 years old with an average of 54.90 (±10.89) years old.

### 2.2. Study Method

All enrolled patients received 3 cycles of PSOX neoadjuvant chemotherapy. The peripheral blood for number and subtypes of CTC and cfDNA measurement were collected before (baseline) and after neoadjuvant therapy (postoperative) on the 10th day after surgery.

### 2.3. Chemotherapy Regimen

PSOX regimen was as follows: paclitaxel of 135 mg/m^2^ and oxaliplatin of 85 mg/m^2^ were injected in the vein on day 1. Tiggio was orally taken based on the patient's body surface area (BSA) from day 1 to day 14, twice a day (2 tablets in the morning and 3 tablets in the evening). 21 days was defined as a chemotherapy cycle. The clinical efficacy and toxicity of neoadjuvant chemotherapy were judged after at least 2 cycles. If the disease progresses during chemotherapy, it will be evaluated after 1 cycle. All cases were confirmed efficacy after 4 weeks.

### 2.4. Circulating Tumor Cell CTC Detection Method (Nanomembrane Filtration and RNA In Situ Hybridization Method)

A total of 10 milliliters (mL) of venous blood from patients before and after chemotherapy and surgery was collected and placed in an ethylenediaminetetraacetic acid (EDTA) anticoagulant tube as a test sample. The samples were centrifugated at 1,500 r/m for 5 minutes within 4 hours, and the plasma phase was removed. CanPatrol® CTC enrichment counting was used to further separate CTC. Furthermore, multiple RNA in situ hybridization technology was used to perform CTC typing detection, and the epithelial type-specific genes (EpCAM, CK8, CK18, and CK19) and the mesenchymal-specific genes (vimentin and twist) were detected, respectively. The amplification probe was hybridized with the above-mentioned type-labeled probe labeled with a fluorescent group to generate a fluorescent signal, the fluorescent signal was read by an automatic identification system, and the CTC typing detection result was automatically judged through the fluorescent signal of different colors.

The CTC results were analyzed. The epithelial CTC was displayed as red fluorescent signal points, and the mesenchymal type was displayed as green fluorescent signal points. The red and green signal points in one cell were displayed as a mixed type.

### 2.5. CfDNA Isolation and Characterization

A total of 10 mL of peripheral venous blood was collected with an EDTA anticoagulation tube. KminTrak plasma extractor was used to extract plasma DNA. Qbit was used to determine the calculated concentration of cfDNA samples. Briefly, the Qbit quantifier reagent and the corresponding amount of DNA quantitative working solution were prepared according to the manufacturer's introduction. Qbit quantitative working solution was divided into QB tubes, and each tube contained 198 microliters (*μ*L). About 2 *μ*L of the extracted nucleic acid was taken and added into the aliquoted working solution, shaken, and mixed well. The standard nucleic acid working solution was used to formulate the standard curve of the Qbit quantifier, and the fluorescence value of the standard curve was about 15,000, and then, the concentration of each nucleic acid was detected. The concentration of each sample was recorded.

### 2.6. Observation Indicators

Chemotherapy efficacy was identified according to the response evaluation criteria in solid tumor 1.1 (RECIST 1.1): (1) complete response (CR): all lesions disappeared and were maintained for 4 weeks; (2) partial response: reduced by 30% in tumor size and were maintained for 4 weeks; (3) progressive disease (PD): 20% increase in tumor size; (4) non-CR/PR/stable disease (SD) before lesions increase; new lesions appear; and (5) SD: based on the minimum sum of the longest diameters after the start of treatment, the reduction was less than the standard for PR, and the increase was not to the standard of PD.

### 2.7. Statistical Analysis

All data were input into spss 20.0 software for statistical analysis. Continuous data were expressed as mean ± standard deviation (Mean ± SD), and an independent sample *t*-test was used for comparison. Categorical data were expressed as examples (%), and the chi-square test was used for comparison. Spearman's correlation coefficient was used to analyze the correlation between CTC number change, cfDNA number change, and chemotherapy effect, and the Kaplan–Meier method was used for survival analysis. *P* < 0.05 indicated that the difference was statistically significant.

## 3. Results

### 3.1. Basic Information of Enrolled Patients

Basic information of 45 patients with AGC is shown in Table 1. Among 45 cases, there were 40 men (88.99%) and 5 women (11.11%). The patients aged more than 55 years old were 17 cases (37.78%), and the patients aged less than 55 years old were 28 cases (62.22%). The pathological typing of all patients was adenocarcinoma. Low, moderate, and high differentiation degrees accounted for 35.55% (16/45), 35.55(16/45), and 28.99 (13/45), respectively. The patients with T I-IV staging were 2 cases (4.44%, stage I-II), 26 cases (57.78%), and 17 cases (37.78%), respectively. All patients with TNM are N + M0.

### 3.2. Baseline Test Results of CTC

**Figure 1:**
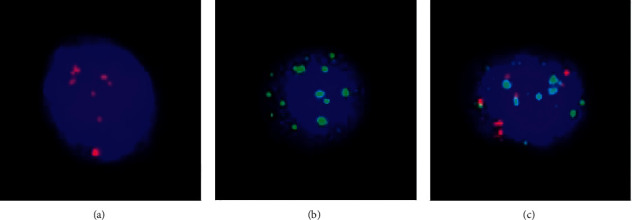
Images of CTCs. (a) Epithelial CTCs; (b) mesenchymal CTCs; and (c) mixed CTCs; CTC, circulating tumor cell.

To investigate the CTC number and subtypes of 45 patients, we identified different CTC characteristics. The result is shown in Figure 1. A total of 87 tests were performed for 45 patients with gastric cancer, and all patients were in the advanced stage. Among them, 14, 14, and 17 patients were tested 3 times, 2 times, and only once, respectively. Neoadjuvant therapy efficacy was as follows: there were 27 patients with neoadjuvant efficacy evaluation results, with 10 PR, 16 stable diseases (SDs), and 1 progressive disease (PD); CTC statistical definition was as follows: when counting the positive rate of mesenchymal CTCs, if we set up mesenchymal CTC = 0, then it was negative and if the mesenchymal CTC ≥ 1, it was positive. Among the 45 patients, 39 were in cTNM stage III and 2 were in cTNM stage IIA. The T staging was used to show the baseline CTC. It can be seen from the above table that according to the tumor T stage stratification, comparing the number of CTC and the positive rate of mesenchymal CTC, it could be seen that the positive rate of mesenchymal CTC in stage 4 patients was higher than that in stage 2-3 patients. The results are shown in Table 2. The above table showed the relationship between the number of peripheral blood CTC and each type and the efficacy of chemotherapy. It could be seen that patients with a small number of mesenchymal CTC were more likely to achieve PR with neoadjuvant treatment.

### 3.3. Correlation between Baseline CTC and Relevant Clinical Indicators of Patients

**Figure 2:**
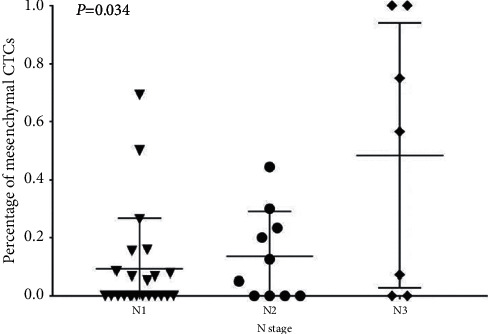
Relationship between mesenchymal CTC percentage and *N* stage. *Y*-axis, mesenchymal CTC percentage; *X*-axis, *N* stage. N1, tumor cells penetrated the second or the third layers of stomach. N2, tumor cells penetrated the second layer of stomach and more distant lymph nodes. N3, tumor cells penetrated the third layer of stomach and more distant lymph nodes.

The clinical information of the enrolled patients with gastric cancer mainly included the following parameters: age, gender, pathological type, tumor location, tumor diameter, degree of differentiation, cTNM staging, T staging, N staging, Lauren type, Borrmann type, whether CA19-9 was normal or not, and so on. The CTC value before neoadjuvant chemotherapy was the baseline CTC. The relationship between CTC and various clinical indicators of gastric cancer was mainly to analyze the total number of CTCs, whether the total number of CTCs >7, the number of each type, and the relationship between the positive/proportion of mesenchymal CTCs and the above parameters. The results of baseline CTC testing are summarized in Table 3 and Figure 2. The results showed that the ratio of mesenchymal CTCs was positively correlated with the N stage, indicating that the *N* stage was larger; the ratio of mesenchymal CTCs was higher. It was consistent with the positive correlation between mesenchymal CTCs and disease stage and prognosis proposed in the current related literature. However, there was no significant correlation between other clinicopathological indicators and the number/type of CTCs.

### 3.4. Correlation between CTCs before Neoadjuvant Treatment (Baseline) and Neoadjuvant Efficacy

CTCs before neoadjuvant chemotherapy were used as the baseline. We compared CTC number and subtype changes after three cycles of neoadjuvant chemotherapy. The efficacy of neoadjuvant treatment was performed according to the RECIST1.1 evaluation standard. There were 27 patients (27/45, 60%) with neoadjuvant efficacy evaluation results, including 10 cases of PR, 16 cases of SD, and 1 case of PD. The results are indicated in Table 4. The above table showed the relationship between the number of peripheral blood CTCs and each type and the efficacy of chemotherapy. It could be seen that patients with a small number of mesenchymal CTC were more likely to achieve PR with neoadjuvant treatment.

### 3.5. Correlation between Clinical Pathology and Neoadjuvant Efficacy

**Figure 3:**
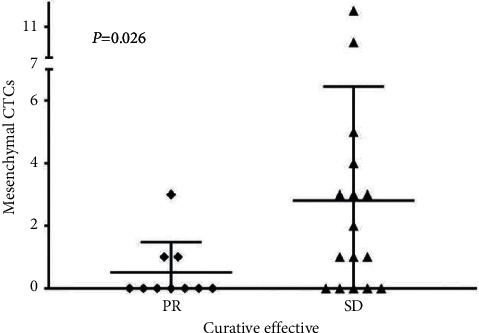
Relationship between mesenchymal CTC number and clinical pathology. *Y*-axis, mesenchymal CTC number; *X*-axis, clinical status. CTCs, circulating tumor cells; PR, partial response; and SD, stable disease.

As shown in Table 5, the age, maximum tumor diameter, pathological stage, and carcinoembryonic antigen (CEA) of the enrolled patients have no correlation with chemotherapy efficacy, but patients with abnormal CA19-9 achieved PR/SD = 0.015 after chemotherapy and PR/SD/PD = 0.018 after chemotherapy (*P* < 0.05). The level of CA19-9 was related to the efficacy of chemotherapy, and patients with positive CA19-9 were more likely to achieve PR with neoadjuvant therapy. Correlation between the end of neoadjuvant therapy (preoperative) CTC and neoadjuvant efficacy was seen that the number/type of mesenchymal CTC after neoadjuvant treatment was significantly related to the efficacy (Figure 3).

### 3.6. Correlation between cfDNA and Clinicopathological Indicators of Gastric Cancer and Neoadjuvant Efficacy

A total of 25 patients in this study were tested for CTCs before neoadjuvant and preoperative chemotherapy, and some patients were tested for cfDNA. Correlation between changes in the number/type of CTCs and changes in cfDNA concentration and neoadjuvant efficacy were analyzed, indicating that the changes in the total number of total CTCs and mesenchymal CTCs were similar in the PR and SD/PD groups, and there was no significant difference (Table 6). As for cfDNA indicators (15 patients had matching cfDNA results before and after neoadjuvant therapy), the trend was that patients in the PR group were more likely to have a decrease/unchanged cfDNA concentration after neoadjuvant therapy (*P*=0.119). The cfDNA concentration at baseline (before neoadjuvant therapy) was not significantly correlated with CTCs and neoadjuvant efficacy; however, the relationship could be seen that the number/type of CTCs after neoadjuvant treatment was not significantly related to the efficacy.

## 4. Discussion

Worldwide, cancer is the second cause of death affecting global residents after cardiovascular disease [22]. Of no doubt, early diagnosis and treatments of cancer are major methods for reducing the death rate in the future. Patients with advanced gastric cancer are still the main group of patients with gastric cancer in China [23] and are the focus of our work. Perioperative chemotherapy is an effective treatment for advanced gastric cancer [24, 25]. However, there is no uniform standard for the evaluation of the efficacy of preoperative chemotherapy [26, 27]. Traditional methods for evaluating the efficacy of gastric cancer treatment or chemotherapy include the following: tumor marker levels, imaging examinations, endoscopic ultrasound before and after treatment, or pathological regression after surgery. Spiral CT is a common method to evaluate the efficacy of chemotherapy for gastric cancer [28], and some studies suggested endoscopy, especially endoscopic ultrasound. A study by Wang et al. [29] pointed out that there was no significant difference in the accuracy of abdominal CT-enhanced scanning and ultrasound gastroscopy in the staging of gastric cancer after neoadjuvant chemotherapy. Considering the intolerance of ultrasound gastroscopy, routine ultrasound gastroscopy is not recommended. Currently, the main biomarkers for gastric cancer diagnosis include CA19-9, CA72-4, and CEA. However, these biomarkers are low specificity. Therefore, many studies explored more reliable and sensitive biomarkers for the early diagnosis of gastric cancer [30, 31].

With the continuous development of molecular technology, liquid biopsy is widely used in the field of tumors. In 2013, it was used as an early cancer detection method [32], which has the advantage of detecting cancer before symptoms appear. The commonly used biomarkers for liquid biopsy include circulating tumor DNA (ctDNA), CTCs, exosomes, and circulating tumor RNA (ctRNA). Currently, only ctDNA and CTC have been approved for clinical use by the FDA [33, 34]. Zhang et al. tested CTC in patients with bladder cancer planned for surgery and found that 44 cases (86.3%) were positive [35]. Many studies revealed that the detection of CTC in patients with colorectal cancer (CRC) [36], nonsmall cell lung cancer (NSCLC) [37], prostate cancer [38], and so on could predict the outcomes of the patients. The clinical findings of the perioperative CTC count and epithelial-mesenchymal transition classification of rectal cancer patients showed that the number of CTC in the peripheral circulation of colorectal cancer patients was reduced, especially for rectal cancer patients who underwent laparoscopic surgery [39]. However, the application of liquid biopsy technology in advanced gastric cancer is limited.

In this study, the total positive rate of CTCs (43/45) in this study was 95.6%, of which the positive rate of mesenchymal type (23/45) was 51.1%. In the study of patients with advanced gastric cancer, the CanPatrol® system monitored the detection rate of CTC capture in the peripheral blood of advanced gastric cancer >80%. The N staging in TNM staging indicates regional lymph node metastasis, but it is difficult to accurately evaluate the N stage before surgery. Generally, high-quality enhanced CT combined with invasive ultrasound gastroscopy is required for assessment, which increases the patient's radiation risk, economic burden, and physical pain. It has been reported that the number of mesenchymal CTC is closely related to the TNM staging and *N* staging of gastric cancer [40]. This study used CTC before neoadjuvant as the baseline, showing that baseline interstitial CTC and *N* staging were significantly correlated (*P*=0.034) and positively correlated. The larger the *N* staging, the proportion of interstitial CTC was the higher. The higher *N* stage indicates that there is cancer cell infiltration in the lymph nodes or lymph vessels around the tumor, and these cancer cells are more likely to enter the peripheral blood system through the lymphatic circulation, which may be the reason why the higher the *N* stage, the easier it is to detect interstitial CTC in the peripheral blood. Therefore, the detection of interstitial CTC at the first diagnosis (before neoadjuvant therapy) may be another indicator for predicting N staging. In TNM staging, the T stage indicates the depth of primary tumor invasion. In the traditional TNM staging method, T staging is of great significance, but T staging reflects the local condition of the tumor, and CTC reflects the peripheral circulation. This may be the reason why T staging is not related to the number of CTCs, and it may also be related to the proportion of T stage in the enrolled patients.

This study found that patients with a small number of intermediate CTC types before neoadjuvant therapy were more likely to achieve PR with neoadjuvant therapy. After analyzing the relationship between the number and classification of CTC before and after neoadjuvant chemotherapy and the efficacy of chemotherapy, the total number of CTCs before and after neoadjuvant therapy was changed, and there was no significant difference in CTC before and after neoadjuvant therapy in patients in the PR, SD/PD, PR, or SD/PD groups. Comparison of changes in the total number of interstitial CTC before and after neoadjuvant therapy is as follows: patients with high interstitial CTC were more likely to have SD/PD (*P*=0.086), but after grouping according to PR and SD/PD, the total number of interstitial CTC before and after neoadjuvant therapy in each group showed no significant difference. It can be seen that the total number of interstitial CTCs was related to the efficacy of neoadjuvant therapy, which was consistent with the conclusions of related studies. The less CTC before neoadjuvant therapy indicated the better effect of neoadjuvant chemotherapy. Therefore, interstitial CTC can be used as an index to predict the efficacy of chemotherapy. This result is consistent with the other reports [41–43] and confirmed that CTC detection is a sensitive and reliable method for the prognosis of patients with AGC.

Normal cell apoptosis will produce cfDNA [44]. The acceleration of cell apoptosis in tumor patients leads to an increase in the number of cfDNA in the peripheral circulation. The tumor burden was greater, and the corresponding cfDNA level was higher. The cfDNA concentration before and after neoadjuvant treatment and the efficacy of chemotherapy were analyzed, and patients with higher cfDNA concentration after neoadjuvant treatment (before surgery) had the lower efficacy of neoadjuvant therapy (SD or PD) (*P*=0.045). Patients in the PR group were more likely to have a decreased/unchanged cfDNA concentration after neoadjuvant therapy (*P*=0.119). Chemotherapy acts on tumors with different cell cycles to prevent tumor cell replication and reduce tumor burden. PR after chemotherapy suggests a reduction in tumor burden in this group of patients through chemotherapy. So the cfDNA concentration of patients was decreased, cfDNA reflected the condition of circulating free DNA, and the concentration did not change, indicating that chemotherapy was effective. On the other hand, the cfDNA before neoadjuvant in the PR group was higher (*P*=0.073), and the cfDNA after neoadjuvant in the SD/PD group was higher. This conclusion was similar to CA-199 and interstitial CTC, and it also reflected from the side that cfDNA concentration before neoadjuvant therapy can predict sensitivity and efficacy of chemotherapy. Comparing the cfDNA concentration before and after neoadjuvant, cfDNA before and after neoadjuvant treatment in the PR group showed a downward trend, but there was no significant difference. The concentration of cfDNA after neoadjuvant in the SD/PD group was significantly increased (*P*=0.008), suggesting that the increase in cfDNA after chemotherapy reflected the poor efficacy of chemotherapy. In summary, the cfDNA concentration before neoadjuvant therapy can predict the efficacy of chemotherapy, and the higher cfDNA concentration before neoadjuvant therapy was relatively sensitive to chemotherapy and easy to achieve PR. If the cfDNA concentration remained elevated after neoadjuvant therapy, it indicated poor chemotherapy efficacy. It was worth noting that the total number of CTC in most patients showed a downward trend 10 days after surgery, but there were also some patients with PR who had an increase in the number of cfDNA after surgery. Follow-up of these patients should be strengthened because their risk of metastasis and recurrence may be higher.

## 5. Conclusions

CTC and cfDNA are safe and minimally invasive detection techniques compared to surgery and endoscopic biopsy. This study suggested that the level of mesenchymal CTC was positively correlated with tumor T staging and N staging, and patients with higher cfDNA concentration before neoadjuvant chemotherapy were easier to achieve PR, indicating that CTC and cfDNA had a certain value in evaluating the efficacy of neoadjuvant therapy for advanced gastric cancer. However, due to the small number of cases currently enrolled in this study, the follow-up has not yet been completed. After a large sample and follow-up study, it may better reflect the role of CTC and cfDNA in the perioperative treatment, recurrence risk assessment, and prediction of the recovery of advanced gastric cancer.

## Figures and Tables

**Table 1:** Basic information of enrolled patients.

	Items	Number (*n*)	Percentage (%)
Gender	M	40	88.89
	F	5	11.11

Age	>55	17	37.78
	≤55	28	62.22

Pathological typing	Adenocarcinoma	45	100
Degree of differentiattion	Low	16	35.55
	Moderate	16	35.55
	High	13	28.89

T staging	1–2	2	4.44
	3	26	57.78
	4	17	37.78

N staging	N0	0	0
	N+	45	100

M, male; F, female; T, tumor; and N, node.

**Table 2:** CTC test data before neoadjuvant therapy (baseline).

T Stage	CTC > 0	Median of CTC	Mean of CTC	Positive rate of mesenchymal CTC
2–3(30)	96.7%(29/30)	111	13.2	46.7%(14/30)
4(15)	93.3%(14/15)	88	10.9	60.0%(9/15)
Total (*n* = 45)	95.6%	110	12.4	51.5%

**Table 3:** Correlation between CTC and patients' clinical indicators before neoadjuvant therapy (baseline).

Spearman's rho			Total CTC	Total CTC(≤7/>7)	Epithelial CTC	Mixed CTC	Mesenchymal CTC	Mesenchymal CTC (≤0/>0)	Mesenchymal CTC proportion
Age (≤60/>60)	*N* = 41	0.2; 31	0.1; 81	0.1; 83	−0.214	0.23; 7	0.171	0.159	−0.231
		0.1; 27	0.2; 34	0.2; 30	0.158	0.11; 8	0.26	0.297	0.127

T stage (≤3/>3)	*N* = 45	r	0.0; 82	0.0; 12	0	0.22; 9	−0.138	0.155	0.126
		P	0.5; 93	0.9; 36	1	0.13; 0	0.365	0.308	0.41

*N* stage	*N* = 40	r	0.2; 32	0.4; 17	0.279	0.02; 5	0.244	0.092	0.134
		P	0.2; 17	0.0; 22	0.136	0.89; 7	0.193	0.63	0.481

CA-199 (normal/abnormal)	*N* = 39	r	0.1; 41	0.0; 36	−0.159	0.08; 4	−0.15	0.133	0.156
		P	0.3; 54	0.8; 13	0.298	0.58; 3	0.325	0.385	0.307

^*∗*^When *P* < 0.05 (two-tailed) or *P* < 0.01 (two-tailed), it indicated a significant correlation; CTC, circulating tumor cells.

**Table 4:** Correlation between CTC before neoadjuvant therapy (baseline) and neoadjuvant efficacy.

Spearman's rho			PR/SD	PR/SD + PD
Total of CTC	*N* = 27	r	0.127	0.069
		p	0.537	0.732

Total of CTC (≤0/>0)	*N* = 27	r	−0.158	−0.217
		p	0.44	0.277

Total of CTC (≤7/>7)	*N* = 27	r	0.22	0.182
		p	0.281	0.364

Epithelial CTC	*N* = 27	r	0.122	0.089
		p	0.553	0.661

Mixed CTC	*N* = 27	r	−0.09	−0.129
		p	0.662	0.522

Interstitial CTC	*N* = 27	r	0.435	0.394
		p	0.026	0.042

Interstitial CTC (≤0/>0)	*N* = 27	r	0.378	0.335
		p	0.057	0.087

Interstitial CTC proportion	*N* = 27	r	0.327	0.292
		p	0.103	0.139

^*∗*^When *P* < 0.05 (two-tailed) or *P* < 0.01 (two-tailed), it indicated a significant correlation. CTC, circulating tumor cell; PR, partial response; SD, stable disease; PD, progressive disease; and *N*, case number.

**Table 5:** Correlation between clinical pathology and neoadjuvant efficacy before neoadjuvant therapy (baseline).

Spearman's rho			PR/SD	PR/SD + PD
Age (≤60/>60)	*N* = 27	r	0.069	0.098
		p	0.734	0.635

T Stage (≤3/>3)	*N* = 27	r	0.217	0.184
		p	0.277	0.367

N stage	*N* = 23	r	0.174	0.157
		p	0.428	0.486

CA19-9 (normal/abnormal)	*N* = 27	r	0.174	0.157
		p	0.428	0.486

^*∗*^When *P* < 0.05 (two-tailed) or *P* < 0.01 (two-tailed), it indicated a significant correlation. CTC, circulating tumor cell; PR, partial response; SD, stable disease; PD, progressive disease; and *N*, case number.

**Table 6:** Correlation between the cfDNA concentration and the efficacy of neoadjuvant at the end of the neoadjuvant therapy (before surgery).

Spearman's rho			PR/SD	PR/SD + PD
cfDNA concentration	*N* = 22	r	0.432	0.405
		p	0.045	0.068

^*∗*^When *P* < 0.05 (two-tailed) or *P* < 0.01 (two-tailed), it indicated a significant correlation. cfDNA, cell-free DNA; CTC, circulating tumor cell; PR, partial response; SD, stable disease; PD, progressive disease; and *N*, case number.

## Data Availability

All data relevant to this study are included within the article and available from the corresponding author upon reasonable request.

## References

[1] Parkin D. M., Bray F., Ferlay J., Pisani P. (2002). Global cancer statistics. *CA: A Cancer Journal for Clinicians*.

[2] González C. A., Sala N., Rokkas T. (2013). Gastric cancer: epidemiologic aspects. *Helicobacter*.

[3] Chen W., Zheng R., Zuo T., Zeng H., Zhang S., He J. (2016). National cancer incidence and mortality in China, 2012. *Chinese Journal of Cancer Research*.

[4] Lim J. S., Yun M. J., Kim M.-J. (2006). CT and PET in stomach cancer: preoperative staging and monitoring of response to therapy. *Radio Graphics*.

[5] Reddavid R., Sofia S., Chiaro P. (2018). Neoadjuvant chemotherapy for gastric cancer. Is it a must or a fake?. *World Journal of Gastroenterology*.

[6] Schuhmacher C., Gretschel S., Lordick F. (2010). Neoadjuvant chemotherapy compared with surgery alone for locally advanced cancer of the stomach and cardia: European Organization for Research and Treatment of Cancer randomized trial 40954. *Journal of Clinical Oncology*.

[7] Cunningham D., Allum W. H., Stenning S. P. (2006). Perioperative chemotherapy versus surgery alone for resectable gastroesophageal cancer. *New England Journal of Medicine*.

[8] Müller Bark J., Kulasinghe A., Hartel G. (2021). Isolation of circulating tumour cells in patients with glioblastoma using spiral microfluidic technology-a pilot study. *Frontiers in Oncology*.

[9] Kolostova K., Pospisilova E., Pavlickova V. (2021). Next generation sequencing of glioblastoma circulating tumor cells: non-invasive solution for disease monitoring. *American Journal of Tourism Research*.

[10] Park C.-K., Oh H.-J., Kim M.-S. (2021). Comprehensive analysis of blood-based biomarkers for predicting immunotherapy benefits in patients with advanced non-small cell lung cancer. *Translational Lung Cancer Research*.

[11] Jiang S.-S., Mao C.-G., Feng Y.-G. (2021). Circulating tumor cells with epithelial-mesenchymal transition markers as potential biomarkers for the diagnosis of lung cancer. *World Journal of Clinical Cases*.

[12] Bade R. M., Schehr J. L., Emamekhoo H. (2021). Development and initial clinical testing of a multiplexed circulating tumor cell assay in patients with clear cell renal cell carcinoma. *Molecular Oncology*.

[13] Guan X., Li C., Li Y. (2021). Epithelial-mesenchymal-transition-like circulating tumor cell-associated white blood cell clusters as a prognostic biomarker in HR-positive/HER2-negative metastatic breast cancer. *Frontiers in Oncology*.

[14] Rink M., Riethdorf S., Yu H. (2020). The impact of circulating tumor cells on venous thromboembolism and cardiovascular events in bladder cancer patients treated with radical cystectomy. *Journal of Clinical Medicine*.

[15] Papadaki M. A., Messaritakis I., Fiste O. (2021). Assessment of the efficacy and clinical utility of different circulating tumor cell (CTC) detection assays in patients with chemotherapy-naïve advanced or metastatic non-small cell lung cancer (NSCLC). *International Journal of Molecular Sciences*.

[16] Lin E., Hahn A. W., Nussenzveig R. H. (2021). Identification of somatic gene signatures in circulating cfDNA associated with disease progression in metastatic prostate cancer by a novel machine learning platform. *The Oncologist*.

[17] Gravina S., Sedivy J. M., Vijg J. (2016). The dark side of circulating nucleic acids. *Aging Cell*.

[18] Peng Y., Mei W., Ma K., Zeng C. (2021). Circulating tumor DNA and minimal residual disease (MRD) in solid tumors: current horizons and future perspectives. *Frontiers in Oncology*.

[19] Casadio V., Calistri D., Salvi S. (2013). Urine cell-free DNA integrity as a marker for early prostate cancer diagnosis: a pilot study. *BioMed Research International*.

[20] Teo Y. V., Capri M., Morsiani C. (2019). Cell-free DNA as a biomarker of aging. *Aging Cell*.

[21] Lafata K. J., Corradetti M. N., Gao J. (2021). Radiogenomic analysis of locally advanced lung cancer based on CT imaging and intratreatment changes in cell-free DNA. *Radiology: Imaging Cancer*.

[22] Sabiu S., Idowu K. (2021). An insight on the nature of biochemical interactions between glycyrrhizin, myricetin and CYP3A4 isoform. *Journal of Food Biochemistry*.

[23] Yang L. (2006). Incidence and mortality of gastric cancer in China. *World Journal of Gastroenterology*.

[24] Sano T., Ichikawa T., Iai A. (2021). [A case of advanced gastric cancer with para-aortic lymph node metastasis with long-term survival by chemotherapy and surgery]. *Gan To Kagaku Ryoho*.

[25] Pelc Z., Skórzewska M., Rawicz-Pruszyński K., Polkowski W. P. (2021). Lymph node involvement in advanced gastric cancer in the era of multimodal treatment-oncological and surgical perspective. *Cancers*.

[26] Tian Y., Wang Q., Wang J. (2021). [Neoadjuvant chemoradiotherapy combined with surgery versus direct surgery in the treatment of Siewert type II and III adenocarcinomas of the esophagogastric junction: long-term prognostic analysis of a prospective randomized controlled trial]. *Zhonghua Wei Chang Wai Ke Za Zhi*.

[27] Miwa K., Oki E., Enomoto M. (2021). Randomized phase II study comparing the efficacy and safety of SOX versus mFOLFOX6 as neoadjuvant chemotherapy without radiotherapy for locally advanced rectal cancer (KSCC1301). *BMC Cancer*.

[28] Liang J.-X., Bi X.-J., Li X.-M. (2018). Evaluation of multislice spiral computed tomography perfusion imaging for the efficacy of preoperative concurrent chemoradiotherapy in middle-aged and elderly patients with locally advanced gastric cancer. *Medical Science Monitor*.

[29] Wang Y., Liu Z., Shan F. (2020). Optimal timing to surgery after neoadjuvant chemotherapy for locally advanced gastric cancer. *Frontiers in Oncology*.

[30] Yao L., Xie Y. (2021). Down-regulation of hsa_circ_0006470 predicts tumor invasion: a new biomarker of gastric cancer. *Journal of Clinical Laboratory Analysis*.

[31] Lee I. S., Ahn J., Kim K. (2021). A blood-based transcriptomic signature for noninvasive diagnosis of gastric cancer. *British Journal of Cancer*.

[32] Fischer J. C., Niederacher D., Topp S. A. (2013). Diagnostic leukapheresis enables reliable detection of circulating tumor cells of nonmetastatic cancer patients. *Proceedings of the National Academy of Sciences*.

[33] Augustus E., Zwaenepoel K., Siozopoulou V. (2021). Prognostic and predictive biomarkers in non-small cell lung cancer patients on immunotherapy-the role of liquid biopsy in unraveling the puzzle. *Cancers*.

[34] Rossi E., Aieta M., Tartarone A. (2021). A fully automated assay to detect the expression of pan-cytokeratins and of EML4-ALK fusion protein in circulating tumour cells (CTCs) predicts outcome of non-small cell lung cancer (NSCLC) patients. *Translational Lung Cancer Research*.

[35] Zhang R., Xia J., Wang Y. (2020). Co-expression of stem cell and epithelial mesenchymal transition markers in circulating tumor cells of bladder cancer patients. *Onco Targets and Therapy*.

[36] Messaritakis I., Sfakianaki M., Vogiatzoglou K. (2020). Evaluation of the role of circulating tumor cells and microsatellite instability status in predicting outcome of advanced CRC patients. *Journal of Personalized Medicine*.

[37] Ntzifa A., Kotsakis A., Georgoulias V., Lianidou E. (2021). Detection of EGFR mutations in plasma cfDNA and paired CTCs of NSCLC patients before and after osimertinib therapy using crystal digital PCR. *Cancers (Basel).*.

[38] Scher H. I., Armstrong A. J., Schonhoft J. D. (2021). Development and validation of circulating tumour cell enumeration (Epic Sciences) as a prognostic biomarker in men with metastatic castration-resistant prostate cancer. *European Journal of Cancer*.

[39] Yin W., Han Y. M., Li Z. L., Huang Z. X., Huang L., Zhong X. G. (2020). Clinical significance of perioperative EMT-CTC in rectal cancer patients receiving open/laparoscopic surgery. *Neoplasma*.

[40] Ning D., Cui K., Liu M. (2021). Comparison of cell search and circulating tumor cells (CTC)-Biopsy systems in detecting peripheral blood circulating tumor cells in patients with gastric cancer. *Medical Science Monitor*.

[41] Moding E. J., Nabet B. Y., Alizadeh A. A., Diehn M. (2021). Detecting liquid remnants of solid tumors: circulating tumor DNA minimal residual disease. *Cancer Discovery*.

[42] Shi Y., Ge X., Ju M., Zhang Y., Di X., Liang L. (2021). Circulating tumor cells in esophageal squamous cell carcinoma - mini review. *Cancer Management and Research*.

[43] Dreyer C. A., VanderVorst K., Free S., Rowson-Hodel A., Carraway K. L. (2021). The role of membrane mucin MUC4 in breast cancer metastasis. *Endocrine-Related Cancer*.

[44] Guan Y., Zhang W., Wang X., Cai P., Jia Q., Zhao W. (2017). Cell-free DNA induced apoptosis of granulosa cells by oxidative stress. *Clinica Chimica Acta*.

